# The Relationship Between Creativity and Intrusive Rumination Among Chinese Teenagers During the COVID-19 Pandemic: Emotional Resilience as a Moderator

**DOI:** 10.3389/fpsyg.2020.601104

**Published:** 2021-01-15

**Authors:** Qian Wang, Xin Zhao, Yuming Yuan, Baoguo Shi

**Affiliations:** Beijing Key Laboratory of Learning and Cognition, School of Psychology, Capital Normal University, Beijing, China

**Keywords:** creativity, intrusive rumination, emotional resilience, teenagers, COVID-19

## Abstract

Coronavirus disease 2019 (COVID-19) has not only resulted in immeasurable life and property losses worldwide but has also impacted individuals’ development, especially teenagers. After the COVID-19 pandemic, individual rumination as an important cognitive process should be given more attention because of its close associations with physical and mental health. Previous studies have shown that creativity as an antecedent variable can predict people’s mental health or adaptation. However, few studies have focused on the relationship between creativity and individual cognitive rumination after traumatic events, and the mechanism underlying this relationship remains unclear. By using the Runco Ideational Behavior Scale (RIBS), the Event Related Rumination Inventory, and the Questionnaire of Adolescent Emotional Resilience, the current study explored the relationship between creativity and intrusive rumination among 1488 Chinese teenagers during the COVID-19 pandemic and analyzed the moderating effect of emotional resilience on the relationship. The results showed that creativity, as assessed by the RIBS, was positively related to teenagers’ intrusive rumination, which implied that a higher level of creative performance could predict more intrusive rumination. Moreover, emotional resilience acted as a moderator in the relationship between creativity and intrusive rumination; the correlation was stronger when emotional resilience was low. These findings provide more evidence of the relationship between creativity and mental health and show the effect of this traumatic event on teenagers.

## Introduction

The rapid spread of the coronavirus disease 2019 (COVID-19) epidemic revealed the virus’ transmissibility and the high associated rates of morbidity and mortality. According to the World Health Organization (WHO), COVID-19 was characterized as a Public Health Emergency of International Concern (PHEIC) on January 30, 2020 (World Health Organization, 2020), and it was classified as a pandemic on March 11, 2020 (World Health Organization, 2020). As of 10:31 am Eastern Standard Time on August 23, 2020, a total of 23,025,622 cases had been confirmed worldwide and 800,420 patients had died, as reported by the WHO (WHO Coronavirus Disease Dashboard, Data last updated: 2020/8/23, 10:31 am CEST).

Generally, epidemics have substantial negative impacts on mental health (Maunder et al., 2003; Ji et al., 2017; Kamara et al., 2017). The COVID-19 pandemic has changed the environment in which people live and has led to increased stress levels (Selvaraj et al., 2020). Numerous news items issued by governments and other national or international agencies, including real and unreal agencies, flooded electronic media (Kumar and Somani, 2020). COVID-19 was reported to have a significant effect on mental health and vice versa (Tandon, 2020). A narrative review of existing articles from the PubMed electronic database showed that subsyndrome mental health problems, such as self-reported stress (8%) and anxiety and depression (16–28%), were commonly found (Rajkumar, 2020). Since the outbreak of COVID-19, several research institutions have studied the psychological impact of Chinese individuals through self-made scales (Qiu et al., 2020; Wang et al., 2020). A cross-sectional study of the general Chinese population was carried out between January 31 and February 2, 2020, with a total sample of 1060 citizens from all provinces in China. The results of SCL-90 questionnaire showed that, due to the virus, more than 70% of the ordinary citizens experienced interpersonal sensitivity (IS), obsessive compulsion (OC), phobic anxiety, insomnia and loss of appetite. In addition, psychological problems such as anxiety, panic, and depression were more likely to appear in minors (Tian et al., 2020).

Faced with the challenges posed by the pandemic, some found the meaning in the suffering and experienced growth, whereas others did not. Many people felt a sense of crisis due to the COVID-19 pandemic, leading to more negative emotions and thoughts, such as intrusive rumination. Intrusive rumination—unconscious thinking about trauma—is characterized by self-reflection (Morrow and Nolen-Hoeksema, 1990) and repeated and passive attention to negative moods (Nolen-Hoeksema et al., 1999; Nolen-Hoeksema, 2000). According to the Response Styles Theory (RST, Nolen-Hoeksema, 1987), rumination is an important susceptibility factor that triggers depression and affects its duration. A large number of empirical studies have confirmed that meditation is related to both depressive episodes and the persistence and aggravation of depressive symptoms (Nolen-Hoeksema et al., 1993; Roelofs et al., 2009). Intrusive rumination is focused on the negative effects of the events and affections related to posttraumatic distress (Wu et al., 2015). In particular, more attention was paid to the negative aspects of life in a passive way after the COVID-19 epidemic, leading to intrusive rumination (Lanciano et al., 2012). Some studies have demonstrated a positive correlation between intrusive rumination and insecure attachment (Caldwell and Shaver, 2012). Zhou et al. (2020) found that intrusive rumination was the mediator between posttraumatic stress disorder (PTSD) and insecure attachment in adolescents. In their research conducted three and one-half years after the Wenchuan earthquake, intrusive rumination was found to have a positive prediction of PTSD. Teenagers who experienced more intrusive rumination exhibited more severe PTSD symptoms (Wu et al., 2015). People who ruminated were more likely to suffer more severe sadness and subsequent depression (Nolen-Hoeksema, 2000). They were also more likely to be creative (Verhaeghen et al., 2005). Another study found that higher reflection scores could predict better creative scores on the RIBS (Cohen and Ferrari, 2010). These studies suggested a link between creativity and intrusive rumination.

### Creativity (RIBS) as the Predictor of Intrusive Rumination

Creativity is an important ability to generate original and divergent thinking and solve problems in a novel and appropriate way (Amabile, 1983; Sternberg and Lubart, 1999). In addition, it is sometimes considered as a personality trait that leads to tactfulness and adaptability. As a complicated construct, creativity involves both cognition and emotion. Csikszentmihalyi (1996) studied the process of creativity and the pleasure of designing or discovering new things, formed by nine elements, including balancing challenges and techniques, removing distractions without fear of failure, and losing self-awareness. The flow concept provided the creator with a state of well-being that supported creativity in moments of adversity as a protective or facilitative factor. Moreover, prior studies have shown that creativity could even be used to create a mental safe space (Desetta and Wolin, 2000). Just as creativity has always been regarded as one of the main driving forces of human civilization and social development, creativity researchers mostly paid attention to the positive meaning of creativity in previous studies. However, with further studies, a new question raised: as an essential tool by which human beings use to understand the world, does creativity result in danger or problems? The dark side of creativity proposed by existing studies offers an affirmative answer and mainly has two aspects. One aspect is the negative consequences for the creative persons themselves (Ludwig, 1992; Silvia et al., 2011), which shows that creative thoughts are accompanied by damage to personal physical and mental health, and a link has been found between mental illness and creativity. For example, Rothenberg (2001) found that manic patients had higher creativity scores. The other significant aspect is the negative consequences for others or society. For instance, creativity may be related to dishonesty, a lack of conscientiousness, offensive behavior, and even criminality (Cropley et al., 2008; Gino and Ariely, 2011; Beaussart et al., 2013). Therefore, exploring the behavior of highly creative individuals during the epidemic is important and necessary.

Compared with the creative personality and process, creative products are easier to quantify. Besides, the assessment of creative products is more reliable (Hennessey, 1994). Therefore, many researchers agree that creativity is often defined by creative products (Guilford, 1957). However, this method has some limitations; for example, it is not well suited for the assessment of teenagers and non-professionals. Although versions of the Wallach Kogan Tests (Wallach and Kogan, 1965), the Torrance Test of Creative Thinking (TTCT, Torrance, 2008), and the Remote Association Test (RAT) by Mednick (1962) have been used in most empirical studies (Urban, 1991), high-quality instruments to measure creativity are still lacking, given that creativity is an indispensable part of the cognitive process. Runco et al. (2001) proposed the daily original, divergent thinking, which could be called daily creativity. They created the 23-item Runco Ideational Behavior Scale (RIBS), a sufficiently reliable assessment for individual and group use. Most of the items described the behaviors in reality, such as actual activities and actions. The discriminant validity of the RIBS was found to be acceptable.

As mentioned above, while creativity results in many benefits, it also relates to the dark side. Previous studies have indicated that people with high levels of creativity had lower latent inhibition scores (Carson et al., 2003), and they often suffer from psychoticism (Eysenck, 1993), substance abuse, suicide (Ludwig, 1995), mood disorders (Kaufman et al., 2007). More studies found that increased creativity was associated with depression, autism, bipolar disorder (Flaherty, 2011), schizophrenia-spectrum disorders, and schizotypal personalities (Vellante et al., 2017). Many creative geniuses have more or fewer dysfunctions in real life, ranging from mental disorders or physical illness to criminal behavior. Examples abound of creative geniuses who also suffered from severe mental illness, such as Van Gogh, Picasso, Tchaikovsky, and Nietzsche. They were all outstanding figures who created great works throughout history, and they all suffered from mental illness at some point during their lives—some even committed suicide (Xu and Shi, 2006). Some findings have also indicated that certain types of mental problems could lead to positive results in creative performance (Dietrich, 2004). For example, two out of five highly creative children were considered to meet the attention deficit hyperactivity disorder (ADHD) criteria (Healey and Rucklidge, 2006). Tolleson and Zeligman (2019) showed that people influenced by Chronic Illness/Disability (CID) had more posttraumatic growth (PTG) experiences. Moreover, the results showed that creativity was a significant predictor of PTG and trauma, which existed together. Shalley et al. (2004) proposed that, regardless of how innovative a person was, when the tasks were finished, the person in charge was non-controlling, supportive, and non-judgmental and provided an environment that limited unnecessary distractions. However, not until the 1990s did researchers accept and explore the dark side of creativity progressively. Since then, studies on the negative side of creativity gradually have a more substantial impact and have attracted more attention. As was previously mentioned, rumination occurs during the COVID-19 pandemic, and it may make individuals pay attention to negative stimuli and lead to negative emotional states. People with higher rumination or reflection scores could perform better on creativity (Verhaeghen et al., 2005; Cohen and Ferrari, 2010). Based on this knowledge, we hypothesized that creativity predicted intrusive rumination during the COVID-19 pandemic (Hypothesis 1).

### Moderating Role of Emotional Resilience

Previous literature has identified resilience as a specific trait or state that helps people recover from negative emotions or events. A study of 241 families across Shanghai and Ji’nan found that family support is relevant to adolescents’ development and emotional resilience. Non-traditional, egalitarian attitudes about parenting help children deal with a changing environment by teaching them innovative strategies (Chang et al., 2011). Since the mid-20th century, researchers have focused on the positive effects of resilience on people experiencing adversity (Rutter, 1987; Luthar et al., 2000). An integrative review of the empirical literature revealed five key themes in resilience (Aburn et al., 2016); in this study, resilience was defined as the capability to quickly adjust negative emotions and successfully overcome a difficult situation. Researchers often regard mental resilience and emotional resilience as the same psychological phenomenon (Denny et al., 2004). Emotional resilience is defined as a self-repairing ability closely related to stress and coping style. In other words, emotional resilience refers to an adaptive mechanism in which individuals can recover quickly from adverse events or negative emotional states and better adapt to the environment (Davidson, 2000).

Numerous studies have confirmed that emotional resilience could help people recover from major adverse events or actively adapt to adversities and contribute to psychological rehabilitation and mental health (Bonanno et al., 2012; Bonanno and Diminich, 2013; Liu et al., 2015). For example, a recent study (Zhang et al., 2020) found that middle school students with high emotional resilience had better learning management skills, more active engagement with studying, and more vital self-regulatory ability when faced with adverse life events. Also, Tranter et al. (2020) found that emotional resilience could mitigate the harmful effects of adverse childhood experiences. As one of the most well-accepted tests used to assess emotional resilience (Zhang and Lu, 2010), the Questionnaire of Adolescent Emotional Resilience is often used in related studies; this questionnaire was used in the current study.

Creativity has long been associated with flexible thinking (Runco and Okuda, 1991), expressiveness, openness (Dollinger et al., 2004), and similar factors of resilient adaptation (Luthar, 2003). Metzl (2009) studied 80 Hurricane Katrina survivors and found that creative thinking ability could predict resilience. The findings showed that flexibility and originality could significantly predict well-being. Precisely, originality could predict extroversion.

In the present study, we hypothesized that emotional resilience could moderate the relationship between creativity and intrusive rumination. There are several reasons to support this idea. Firstly, according to Everall et al. (2006), emotional resilience is considered a stabilizing personality trait or ability that protects individuals against adversity and risk. This stabilizing characteristic is strongly related to cognitive functions, such as an excellent problem-solving ability (Dumont and Provost, 1999; Aburn et al., 2016). Besides, many researchers have found that creativity is closely related to emotional resilience (Runco and Richards, 1998; Metzl and Morrell, 2008; Metzl, 2009); Wolin and Wolin (1993) suggested that creativity is a type of resilience. Resilient thinking, such as creative thinking, contributes to solving problems in unique ways with the existing resources (Hartling, 2005).

Secondly, teenagers may have frequently received and reprocessed negative information during the COVID-19 pandemic, leading to depression. Given the suddenness and severity of the epidemic, young individuals may not know and can deal with the situation. The more they think, the more intrusive rumination affects their feelings and mental health. However, the most essential characteristic of emotional resilience is that it helps people who endure lasting depression generate positive emotions (Tugade et al., 2005). It can also reduce intrusive rumination (Li et al., 2018). In addition, according to the Dual Pathway Model proposed by De Dreu et al. (2008), this hedonic tone improves teenagers’ creativity by facilitating a higher level of cognitive flexibility. Thus, adolescents with high degrees of emotional resilience may be more flexible concerning coping with negative situations and may be able to turn negative information into positive thoughts.

Finally, in a recent study, Liang et al. (2020) found that resilience moderated the relationship between creative thinking and posttraumatic stress symptoms among Chinese adolescents exposed to the Lushan earthquake, which supports the idea that emotional resilience can be conducive to alleviating the negative impact of the pandemic. Although a series of studies have explored the mechanisms underlying the relationship between creativity and intrusive rumination, few studies have examined the moderating role of emotional resilience. Based on this theoretical and empirical foundation, we propose that emotional resilience moderates the relationship between creativity and intrusive rumination (Hypothesis 2).

### The Present Study

Teenagers in junior high school are in a transitional period from immaturity to maturity, during which their physiology and psychology are rapidly changing. Moreover, their psychological development is precarious. Therefore, when they encounter setbacks and difficulties in life, they are more likely to be confused and helpless; some may even have serious psychological problems. However, we also found that some individuals could quickly recover from negative emotions when faced with similar setbacks without suffering physical or mental damage. In contrast, others experienced negative emotions and could not extricate themselves, leading to psychological and behavioral problems. A small number of people even exhibit excessive behaviors. For example, during this epidemic, individuals with different creativity levels have experienced various degrees of intrusive rumination. Why does this happen? Answering this question involves addressing the issue of individual emotional resilience. Few studies exist on this topic, and our analysis was performed to enrich this research area.

The current study examined the relationship between creativity and intrusive rumination among Chinese teenagers after the outbreak of COVID-19. In particular, we established a moderation model to explore whether emotional resilience plays a moderating role in the association between creativity and intrusive rumination.

## Materials and Methods

### Participants

Participants were 1488 7th and 8th-grade students in two schools from one Province in Central China, including 653 girls and 835 boys. A total of 71.3% of the sample was from an urban school, and 28.7% was from a rural school. The distribution of participants in the current study is detailed in Table 1. All participants were asked to provide sufficient information, including information on demographic variables, creativity (RIBS), intrusive rumination, and emotional resilience. The students’ average age was 13.85 (*SD* = 0.891 years, range = 12–16) years.

**TABLE 1:** Demographic distribution of the participants.

	Male		Female		Total
		
	Urban	Rural	Urban	Rural	
7th Grade	292	141	242	80	755
8th Grade	289	113	238	93	733
Total	581	254	480	173	1488

### Measures

#### Creativity

Students’ creativity was assessed using the RIBS, which comprises 23 items (Runco et al., 2001). This reliable scale was based on the theory that creative ideation should lead to novel and original products, and was designed to measure people’s tendency to generate creative ideas, called creative thinking. Students responded on a five-point Likert response scale ranging from one (strongly disagree) to five (strongly agree). Sample items are “I come up with a lot of ideas or solutions to problems,” “I am able to think about things intensely for many hours,” and “I often have trouble sleeping at night because so many ideas keep popping into my head.” The questionnaire has been used in China and has been shown to have good reliability and validity (e.g., O’Neal et al., 2015; Yang et al., 2020). The scale showed remarkable reliability in the current study, with a Cronbach’s α coefficient of 0.94.

#### Intrusive Rumination

Intrusive rumination was assessed with the revised version of the Event Related Rumination Inventory (ERRI, Wu et al., 2015). This test was derived from the original ERRI (Cann et al., 2011). The score for each item ranges from zero points (never) to three points (always). Higher scores indicated a stronger tendency to ruminate. The questionnaire has been successfully used to predict rumination among adolescents after an earthquake (see Zhou et al., 2017, for an in-depth review of the reliability and validity). In the present study, only the first ten items about intrusive rumination were used. Also, the phrase “After the earthquake” was changed to “After the COVID-19 epidemic.” Sample items included “I find myself thinking about it spontaneously,” “Something else will remind me of this experience,” and “The thought of this experience distracts me from concentrating.” In the current study, the scale showed excellent reliability, with a Cronbach’s α coefficient of 0.91.

#### Emotional Resilience

The Questionnaire of Adolescent Emotional Resilience (Zhang and Lu, 2010) was used to assess students’ emotional resilience during the COVID-19 pandemic. Students responded on a six-point response scale ranging from one (strongly disagree) to six (strongly agree), with higher scores indicating higher levels of ability to cope with negative emotions. A sample item is “I quickly get over and recover from negative emotions.” The questionnaire has also been used by others and has been shown to have good reliability (0.80) and validity (0.83) (Tong, 2018). The scale showed acceptable reliability in the current study, with a Cronbach’s α coefficient of 0.71.

### Procedure

Data were voluntarily provided in May 2020 through a web-based survey that lasted approximately 10–15 min. Students finished this survey. Their responses were uploaded onto an online survey platform used to collect survey data; the survey was completed either in a computer class or via their parents’ WeChat. The research respondents were guaranteed anonymity. The present research was approved by the Research Ethics Board of Capital Normal University. All participants and their parents provided informed consent before participating in the survey.

### Data Analyses

SPSS 23.0 and PROCESS macro 3.3 for SPSS were used to analyze the data. First, we computed descriptive statistics to assess the demographic characteristics. Second, we used model 1 (Hayes, 2013) of the PROCESS macro for SPSS to test the moderating effect of emotional resilience on the relationship between the RIBS score and intrusive rumination. The bootstrapping method was used, and the resultant 95% confidence intervals of 5000 resamples of the data were inspected.

## Results

### Common Method Bias Test

Because all variables were self-reported by the respondents, common method bias may have existed (Podsakoff et al., 2003). Therefore, we examined this issue using the Harman single-factor test. The approach is to combine all the variable measurement items in unrotated factor analysis. If only one factor remains or the first factor explains the vast majority of the variation, there is severe common method bias. Conversely, if those criteria are not met, there is no serious common method bias. The study employed this method. A total of 7 factors were obtained and together explained 57.05% of the variance. The proportion of the variation explained by the first principle component was 24.18%, which did not exceed the critical value (40%). Thus, common method bias did not severely affect this study.

### Descriptive Statistics

The correlations, means, and standard deviations of the research variables are presented in Table 2. As expected, intrusive rumination was positively related to creativity (RIBS) (*r* = 0.21, *p* < 0.01) and emotional resilience (*r* = −0.14, *p* < 0.01). As shown in Table 3, further analysis indicated that male students (*M* = 39.02, *SD* = 9.05) scored lower than females (*M* = 41.45, *SD* = 7.92) on emotional resilience [*t*_(1486)_ = −5.43, *p* < 0.001], and female students (*M* = 9.38, *SD* = 6.11) scored higher than males (*M* = 8.61, *SD* = 5.71) on intrusive rumination [*t*_(1486)_ = −2.47, *p* < 0.05]. However, no gender difference was found regarding creativity [*t*_(1486)_ = −0.01, *p* = 0.993]. No grade difference was found regarding emotional resilience [*t*_(1486)_ = 0.06, *p* = 0.951], intrusive rumination [*t*_(1486)_ = 1.39, *p* = 0.163], or creativity [*t*_(1486)_ = −1.37, *p* = 0.171].

**TABLE 2:** Means (*M*), standard deviations (*SD*), and correlations between variables.

Variables	Mean (*SD*)	1	2
(1) RIBS	71.41 (16.44)		
(2) Intrusive Rumination	9.04 (5.95)	0.21**	
(3) Emotional Resilience	40.38 (8.52)	0.18**	−0.14**

*N* = 1488, **p < 0.01.

**TABLE 3:** Differences in the RIBS scores, intrusive rumination, and emotional resilience stratified by gender.

	Total (*N* = 1488)	Male (*n* = 835)	Female (*n* = 653)			
			
	*M* (*SD*)	*M* (*SD*)	*M* (*SD*)	*t*	*p*	*Cohen’s d*
(1) RIBS	71.41 (16.44)	71.41 (15.41)	71.42 (17.22)	–0.01	0.993	< 0.01
(2) Intrusive Rumination	9.04 (5.95)	8.61 (5.71)	9.38 (6.11)	–2.47	0.013	0.13
(3) Emotional Resilience	40.38 (8.52)	39.02 (9.05)	41.45 (7.92)	–5.43	< 0.001	0.29

### Testing for a Moderating Effect

**FIGURE 1:**
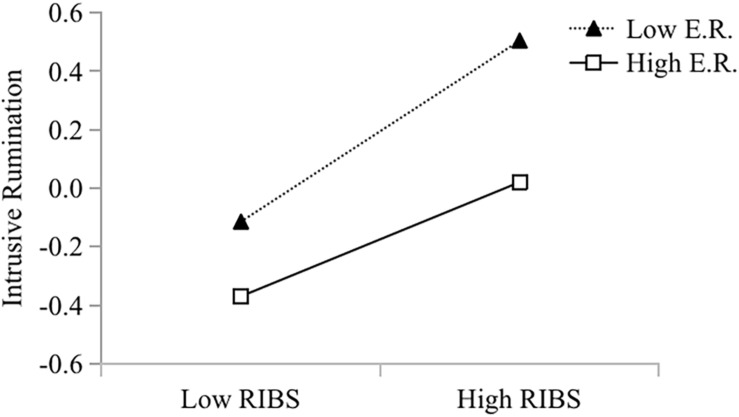
Rumination as a function of RIBS and emotional resilience. Functions are graphed for two levels of emotional resilience: 1 standard deviation above the mean (High E.R.) and 1 standard deviation below the mean (Low E.R.). Standardized values of RIBS and emotional resilience were used in all inferential analyses. E.R., emotional resilience.

To examine the moderation hypothesis, this study using the PROCESS macro (Model 1) to estimate the model parameters. Intrusive rumination, emotional resilience, and their interaction term (RIBS × emotional resilience) were entered into the model, and we standardized the scores for these three scales. Gender, age, and grade were included as covariates to control their influence on the results. As shown in Table 4, there was a main effect of creativity (RIBS) on intrusive rumination (β = 0.25, *p* < 0.001), and this effect was moderated by emotional resilience (β = −0.06, *p* = 0.01). The further simple regression analysis (Table 5) showed that the 95% confidence intervals did not include a zero. The association between creativity (RIBS) and intrusive rumination was stronger for students with low levels of emotional resilience (*b*_*simple*_ = 0.30, *p* < 0.001) than for students with high levels of emotional resilience (*b*_*simple*_ = 0.19, *p* < 0.001). Our study separately plotted low and high levels of emotional resilience (one *SD* below the mean and one *SD* above the mean), as shown in Figure 1. Emotional resilience weakened the effect of RIBS on intrusive rumination.

**TABLE 4:** Test of the moderating effect of emotional resilience on the relationship between creativity (RIBS) and intrusive rumination.

Predictors	Intrusive Rumination					
	
	β	*SE*	*t*	*p*	Lower	Upper
*Gender*	0.18	0.05	3.65	0.003	0.09	0.28
*Age*	0.05	0.03	1.52	0.129	–0.01	0.11
*Grade*	–0.13	0.06	–2.28	0.023	–0.24	–0.02
RIBS	0.25	0.03	9.97	< 0.001	0.20	0.30
Emotional Resilience	–0.18	0.03	–7.10	< 0.001	–0.24	–0.13
RIBS × E.R.	–0.06	0.02	–2.58	0.010	–0.10	–0.01
*R*^2^	0.09					
*F*	25.06***					

*N* = 1488. E.R., emotional resilience. ***p < 0.001.

**TABLE 5:** Conditional effects of creativity (RIBS) at specific levels of emotional resilience.

Conditional effect of E.R.	Estimate	*S.E*	*t*	*p*	95% CI	
					
					Lower	Upper
*-1 SD*	0.31	0.34	8.89	<0.001	0.24	0.38
*M*	0.25	0.25	9.97	<0.001	0.20	0.30
*+1 SD*	0.19	0.32	6.01	<0.001	0.13	0.26

*E.R., emotional resilience.*

## Discussion

The present study aims to explore the relationship between creative ideational behavior and intrusive rumination and the effect of emotional resilience as a potential moderator of the relationship. The results suggested that creative ideational behavior was positively associated with intrusive rumination during the COVID-19 pandemic. Specifically, the relationship was more robust when teenagers’ level of emotional resilience was low. These findings provide more evidence of the links between creativity and mental health, and show the effect of the COVID-19 pandemic as a traumatic event on teenagers.

### The Positive Association Between Creativity and Rumination

It is generally accepted that creativity has both benefits and challenges (Kapoor and Khan, 2017). The dark side of creativity has become an essential topic in creativity research, which has focused on aggressive behavior, unethical conduct, and crime or terrorism (Cropley, 2010). Researchers even found more problems with dishonesty than positive outcomes resulting from creativity (Gino and Ariely, 2011), which means people with high levels of divergent thinking or more creative ideational behavior can generate more negative thoughts, including more lies (Walczyk et al., 2008). Besides, good creative potential can predict high sensitivity and mood disorders (Ludwig, 1995). As far, supporting evidences from a lot of studies have demonstrated that highly creative people may be more likely to suffer from mental illness (Sass, 1992; Claridge and McDonald, 2009; Fisher, 2015). To explain this phenomenon, we should consider the role of rumination.

Based on the definition of Cann et al. (2011), intrusive rumination is “an uninvited invasion” into one’s cognitive world. It involves thinking about experiences that one does not want to think about. Recent studies have shown that the COVID-19 pandemic has negatively impacted many components of students’ development, including their stress (Bao et al., 2020), anxiety, and depressive disorders (Xiang et al., 2020). During and after the pandemic, teenagers experienced many negative emotions due to the existence of many rumors and a sense of uncertainty (Wang and Zhao, 2020). Therefore, they are more likely to ruminate on the pandemic event. According to Response Style Theory, they could also become anxious and stressed if they continually repeat negative thoughts and passively focus on responding to negative moods (Nolen-Hoeksema, 1991). On the other hand, creativity reflects tolerance and response to new environmental stimuli. Individuals with high creativity may be more sensitive and susceptible to the pandemic event and more strongly affected by negative news during the pandemic, and produce more rumination including the intrusive one. According to Verhaeghen et al. (2005), rumination was related to depressive symptoms, creative interest, creative fluency, originality, and elaboration. Cohen and Ferrari (2010) revealed that reflective rumination could significantly predict creativity in terms of RIBS.

To sum up, the result of this study showed that creative ideational behavior could predict intrusive rumination, which suggested that creative teenagers were more vulnerable to the psychological effects of traumatic events like the COVID-19 pandemic and experienced more intrusive rumination. This result may be since creativity attaches importance to liberal fantasy or association. For example, Drus et al. (2014) found students with a high level of divergent thinking exhibited more liberal response bias for negative information, which might lead to more intrusive rumination. Besides, a recent study suggests that people with high levels of divergent thinking experience more mind wandering, which leads to more negative emotions (Yamaoka and Yukawa, 2020). However, it is noteworthy that this study’s positive relationship did not support the results of previous studies in which intrusive rumination was observed to interfere with the problem-solving process (Watkins and Brown, 2002; Watkins and Moulds, 2005). One possible explanation is that the relationship between creativity and rumination may vary according to individual and environmental factors. In prior studies on rumination, both the selection of subjects and the measurement were carried out in a safe environment. In contrast, this study was conducted during the epidemic, and the results were more reflective of the real-world situation. Nevertheless, more empirical studies are needed in the future to explore the mechanism underlying the relationship between creativity and rumination in different situations.

### The Moderating Role of Emotional Resilience

Many researchers have pointed out that creativity is closely related to mental resilience (Kim, 2015; Chen and Padilla, 2019; Xu et al., 2019). High trait resilience was found to predict more creative thinking (Liang et al., 2020). Creative thinkers are more likely to change their minds and use multiple approaches to solve problems rather than give up, further improving their mental resilience (Seale et al., 2013). This study showed a similar result that emotional resilience was positively correlated with teenagers’ ideational behavior. To explain the link between creativity and resilience, Metzl and Morrell (2008) put forward a model suggesting that both creative thinking and creative personality are conducive for recovering from adversity. Our research further expands this model by introducing creative ideational behavior. Moreover, Metzl (2009) indicated that originality and flexibility seem to be the most common cognitive pathways to emotional resilience. Thus, the positive relationship between creativity and emotional resilience can be understood as having a mutually reinforcing effect during the pandemic.

Hypothesis testing revealed a moderating role of emotional resilience in the current study. Specifically, emotional resilience reduced the predictive power of creativity for intrusive rumination, and teenagers with higher emotional resilience were less likely affected by the negative effects of creativity. Many previous studies have indicated that emotional resilience is a vital protective factor that helps people cope with traumatic experiences and maintain mental health (Pearson, 2007; Bonanno and Mancini, 2012; Bonanno et al., 2012; Bonanno and Diminich, 2013). According to the resilience strategy model, teenagers’ resilience can be a positive factor that allows them to overcome adversities without suffering long-term adverse effects (Hunter and Chandler, 1999). Block and Kremen (1996) suggested that people with high degrees of emotional resilience are more likely to experience positive emotions and recover quickly from stressful and negative emotional experiences, making them more flexible and adapting to the current environment. According to the Dual Pathway Model proposed by De Dreu et al. (2008), positive emotions could involve increased cognitive inclusiveness and flexibility that promotes creativity. Also, Fredrickson (2003) pointed out that individuals with more positive emotions had more creative thinking. These studies have shown that creativity is closely related to emotional resilience. Based on this, it is reasonable to speculate that a highly creative individual may also have higher emotional resilience.

On the other hand, the present study found a significant negative relationship (*r* = −0.14, *p* < 0.01) between emotional resilience and intrusive rumination, similar to prior studies. For example, Li et al. (2018) found that emotional resilience was negatively related to intrusive rumination. Considering that intrusive rumination is associated with poor mental health, this result suggests that emotional resilience is related to teenagers’ positive development. According to previous studies, middle school students who had low emotional resilience were found to be inclined to narrow the focus of their thoughts (Schiffrin and Falkenstern, 2012). While, teenagers with high emotional resilience may reverse the negative effect of the COVID-19 pandemic by transforming the given information, adjusting their emotional state, and reshaping their cognitive process, facilitating positive adaptation. This is precisely the moderating effect found in this study. It is also consistent with the findings of Masten (2001) regarding the resilience of teenagers after a natural disaster, which indicates that resilience could be more important than coping ability or creativity for mental health. These conclusions imply that we should routinely cultivate students’ mental resilience at school. Additionally, psychological aid workers could reduce the damage caused by intrusive rumination by increasing an individual’s mental resilience.

### Implications and Limitations

This study provides insights into the psychological state of students coping with the COVID-19 pandemic. Previous studies often focused on the positive effects of creativity and emphasized the need to enhance creativity. Our research has focused on the other side of creativity: individuals with high levels of creativity are more susceptible to the mental effects of catastrophic events and produce more negative thoughts. This finding provides us with a deeper understanding of how highly creative individuals are affected by traumatic events. Additionally, our findings have important practical implications. Firstly, teachers must pay more attention to the mental health of highly creative adolescents. Teenagers with high levels of creativity tend to have more potent imagination abilities; thus, they can perform well in a safe environment but not as well in an unsafe environment. Teachers and parents should take action to reduce their negative fantasy. Second, as we pay increasing attention to the cultivation of students’ creativity, this study shows that teachers and school administrators should also be trained in psychological adaptability to ensure that students can cope with stressful events in the future. Besides, our study found that the relation between creativity and intrusive rumination was more substantial in students with lower levels of emotional resilience. Emotional resilience is an important buffer that can reduce adolescents’ negative emotions, which is consistent with prior research results (Wang et al., 2018). Moreover, other personal and contextual boundary conditions might affect the relationship between creativity and intrusive rumination. Future studies should analyze the moderating role of mindfulness in this relationship since previous studies have revealed its connection with increased cognitive adjustment at work and creativity (Montani et al., 2019, 2020).

In addition, several limitations should be noted. Firstly, because this was a cross-sectional study, we could not make causal inferences about the results or investigate the dynamic process. Secondly, all of the data were obtained with a self-reported scale, and the validity may have been affected by social desirability and other biases. Future studies could use multiple data points from different informants (e.g., peers, parents, and teachers) or utilize a longitudinal design to examine the relationship among the RIBS, intrusive rumination, and emotional resilience. Lastly, we used a convenience sampling method, and all teenagers were recruited from two schools in the same area. Because the severity of the pandemic has varied in different regions, the sample population’s representativeness in this study is limited. Future studies should compare other groups from different school levels (e.g., primary versus high schools), diverse types of schools (private versus public), and other regions and countries.

## Conclusion

In summary, the present study shows that creativity can be a risk factor for adolescent intrusive rumination under the influence of crisis such as the COVID-19 pandemic. Furthermore, the moderation analysis revealed that emotional resilience buffers the relationship between creativity and intrusive rumination, with a weaker effect between creativity and intrusive rumination among adolescents with a high level of emotional resilience. Our findings shed light on the psychological problems that highly creative people may encounter in stressful situations and provides a new perspective on creativity and mental health.

## Data Availability Statement

The raw data supporting the conclusions of this article will be made available by the authors, without undue reservation.

## Ethics Statement

The studies involving human participants were reviewed and approved by the Research Ethics Board of Capital Normal University. Written informed consent to participate in this study was provided by the participants’ legal guardian/next of kin.

## Author Contributions

BS designed the study and performed the investigation. QW, XZ, YY, and BS analyzed the data and wrote the manuscript. All the authors contributed to the article and approved the submitted version.

## Conflict of Interest

The authors declare that the research was conducted in the absence of any commercial or financial relationships that could be construed as a potential conflict of interest.
